# Social Networks of Lexical Innovation. Investigating the Social Dynamics of Diffusion of Neologisms on Twitter

**DOI:** 10.3389/frai.2021.648583

**Published:** 2021-11-01

**Authors:** Quirin Würschinger

**Affiliations:** Department of English and American Studies, LMU, Munich, Germany

**Keywords:** lexicology, lexical innovation, sociolinguistics, diffusion, social media, Twitter, time-series analysis, social network analysis

## Abstract

Societies continually evolve and speakers use new words to talk about innovative products and practices. While most lexical innovations soon fall into disuse, others spread successfully and become part of the lexicon. In this paper, I conduct a longitudinal study of the spread of 99 English neologisms on Twitter to study their degrees and pathways of diffusion. Previous work on lexical innovation has almost exclusively relied on usage frequency for investigating the spread of new words. To get a more differentiated picture of diffusion, I use frequency-based measures to study temporal aspects of diffusion and I use network analyses for a more detailed and accurate investigation of the sociolinguistic dynamics of diffusion. The results show that frequency measures manage to capture diffusion with varying success. Frequency counts can serve as an approximate indicator for overall degrees of diffusion, yet they miss important information about the temporal usage profiles of lexical innovations. The results indicate that neologisms with similar total frequency can exhibit significantly different degrees of diffusion. Analysing differences in their temporal dynamics of use with regard to their age, trends in usage intensity, and volatility contributes to a more accurate account of their diffusion. The results obtained from the social network analysis reveal substantial differences in the social pathways of diffusion. Social diffusion significantly correlates with the frequency and temporal usage profiles of neologisms. However, the network visualisations and metrics identify neologisms whose degrees of social diffusion are more limited than suggested by their overall frequency of use. These include, among others, highly volatile neologisms (e.g., *poppygate*) and political terms (e.g., *alt-left*), whose use almost exclusively goes back to single communities of closely-connected, like-minded individuals. I argue that the inclusion of temporal and social information is of particular importance for the study of lexical innovation since neologisms exhibit high degrees of temporal volatility and social indexicality. More generally, the present approach demonstrates the potential of social network analysis for sociolinguistic research on linguistic innovation, variation, and change.

## 1 Introduction

Societies continually evolve, new products and practices emerge, and speakers coin and adopt new words when they interact and share information. How do these new words spread in social networks of communicative interaction?

In a recent paper analysing contagion patterns of diseases in *Nature Physics*, Hébert-Dufresne et al. (2020) suggest that the spread of viruses like SARS-CoV-2 follows principles of complex contagion through social reinforcement, and that it matches the dynamics of diffusion of cultural and linguistic innovations such as new words and internet memes. Does this confirm the widespread perception that new words ‘go viral’? Influential sociolinguistic models of the spread of linguistic innovations like the S-curve model (Milroy 1992) share fundamental features with earlier economic models of diffusion (Rogers 1962). It is often assumed that diffusion in social networks follows universal trajectories and that rates of spread depend on social dynamics such as network density and the presence or absence of weak ties (Granovetter 1973). Unlike research on biological and cultural diffusion processes, however, sociolinguistic research has only recently been provided with data sources that are equally suitable for large-scale, data-based approaches which can rely on network analyses to study these phenomena empirically.

Social media platforms like Twitter have changed the way we communicate and how information spreads, and they offer valuable data for empirical research. For linguists, social media provides large amounts of data of authentic language use which opens up new opportunities for the empirical study of language variation and change. The size of these datasets as well as their informal nature allow for large-scale studies on the use and spread of new words, for example, to gain insights about general trajectories of diffusion (Nini et al., 2017) or about factors that influence whether new words spread successfully (Grieve, 2018). Moreover, metadata about speakers facilitate the study of aspects of diffusion that go beyond what can be captured by usage frequency alone. Recent work has used Twitter data to investigate the geographical spread of lexical innovations (Eisenstein et al., 2014; Grieve et al., 2016), for example.

Data about the communicative interaction of speakers additionally allows performing network analyses of the social dynamics of diffusion processes. Network science approaches to social media data have been successfully employed in diverse fields, for example, to study the spread of diseases (Lu et al., 2018), opinions (West and Hristo, 2014) and political attitudes (Pew Research Center 2019). While the study of social networks has a long research tradition in sociolinguistics and has shaped influential models of diffusion (e.g., Milroy and Milroy 1985), large-scale network analyses of sociolinguistic phenomena have only recently become more widespread. These new data sources and methodological advances put computational sociolinguistics in an excellent position to gain new insights and to test long-standing theoretical models empirically.

In the area of lexical innovation, this can serve to evaluate important theoretical concepts like the role of early adopters, network density and weak ties in the diffusion of new words. For example, previous approaches have used computational modelling to test the validity of the S-curve model (Blythe and Croft 2012), and to model processes of simple and complex contagion of linguistic innovations in social networks (Goel et al., 2016). Applying social network analysis to bigger samples of neologisms and tracking their use and spread on social media datasets promises to provide a more detailed picture of social diffusion. Social network information has the potential to more accurately assess the degrees to which the adoption of new words remains limited to closely connected sub-communities or whether they reach larger parts of the speech community.

This paper aims to explore the role of network information and temporal dynamics in assessing the diffusion of lexical innovations on Twitter. I use several quantitative and qualitative methods to study diffusion. I conduct a longitudinal study monitoring the use of a broad sample of neologisms to analyse their usage frequency and the temporal dynamics underlying their use. Next, I use social network analyses to get a better picture of the sociolinguistic dynamics at play, to assess different pathways and overall degrees of diffusion. Lastly, I combine both approaches to get a more detailed picture of the diffusion of the neologisms in the sample, and to assess the results of both approaches to diffusion.

The paper is structured as follows. Section 2 introduces the theoretical framework for modelling and measuring the diffusion of lexical innovations which forms the basis for the empirical study. Section 3 presents information about the sample of neologisms and the Twitter dataset this study is based on. Section 4 describes the methods used for analysing diffusion. Section 5 presents the results of the empirical study. I analyse diffusion on the basis of frequency and social networks and integrate the results obtained from both approaches. Section 6 summarises and discusses the results from the empirical study and draws implications about the role of frequency and network-based measures for the study of diffusion.

## 2 Modelling and Measuring the Diffusion of Lexical Innovations

### 2.1 Modelling Diffusion

Neologisms are on a continuum from entirely novel word-formations to fully established lexemes which are familiar to the majority of the speech community. Neologisms have spread to some extent, but are still perceived as new or unknown by many speakers (Schmid 2016). On one end of the continuum, ‘ad-hoc formations’ are new words that have been coined in a concrete communicative situation, but are not adopted by interlocutors and do not diffuse beyond their original usage contexts (Hohenhaus 1996). On the other end, fully established words are known and used by the majority of the speech community. Neologisms occupy an intermediate position between both poles and can be defined as ‘(…) lexical units, that have been manifested in use and thus are no longer nonce-formations, but have not yet occurred frequently and are not widespread enough in a given period to have become part and parcel of the lexicon of the speech community and the majority of its members’ (Kerremans 2015, 31).

Diffusion can be seen as the process that transports successful neologisms along this continuum while they are becoming increasingly conventional in the speech community. The S-curve model (Milroy 1992; Nevalainen 2015; Labov 2007) expects an S-shaped trajectory for the spread of linguistic innovations and makes specific assumptions about the sociolinguistic characteristics of speakers involved in the diffusion process. In a first stage of slow diffusion, only a small number of early adopters take up the innovative words. These individuals typically form dense networks which are connected by strong ties. In the case of successful diffusion, the initial stages are followed by an acceleration in spread when new words increasingly reach speakers outside the initial communities. Weak ties (Granovetter 1973) play an important role in allowing the innovations to reach a bigger parts of the speech community. During later stages, rates of diffusion slow down again as the majority of the speech community has already adopted the new words, while a minority of speakers remains resistant to take up the new words.

The Entrenchment-and-Conventionalization Model (Schmid 2020) conceptualises the conventionalization of linguistic innovations as involving two processes: usualization and diffusion. Diffusion is defined as the process that ‘brings about *a change in the number of speakers and communities* who conform to a regularity of co-semiotic behaviour and a change in the conformity regarding the types of cotexts and contexts in which they use it.’ (Schmid 2020, 178–179, emphasis mine) In the case of a given new word, it is coined by an individual speaker and first reaches a community of speakers who might be closely-connected to the coiner and/or share interests related to the given neologism. With more advanced diffusion, the word spreads to larger numbers of speakers and increasingly also becomes conventional in other communities of speakers. The process of usualization, by contrast, leads to the increasing establishment of a given neologism by repeated use within one community of speakers. Neologisms thus show high degrees of conventionality, when they exhibit high usage intensity across a large number of speakers and communities.

### 2.2 Measuring Diffusion

Earlier empirical work on lexical innovation had to rely on smaller, general-purpose linguistic corpora. The low-frequency nature of neologisms limited earlier studies to conducting case studies on selected neologisms (Hohenhaus 1996) or on specific domains of neology (Elsen 2004). In recent years, research on lexical innovations has seen an upsurge in large-scale empirical investigations on the diffusion of neologisms, thanks to the availability of new data sources and computational methods.

The increasing availability of web corpora significantly extended the opportunities for large-scale corpus analyses. Modern corpora like the NOW corpus (Davies 2013) allow to study more comprehensive samples of neologisms and enable researchers to monitor their use over time, which is essential for investigating diffusion processes. In addition to general-purpose web corpora, several research groups built dedicated tools and specialized corpora for the monitoring and analysis of neologisms (Renouf et al., 2007; Kerremans et al., 2012; Lemnitzer, 2010; Gérard et al., 2017; Cartier 2017).

More recently, social media data have become an increasingly important alternative to web corpora. Language use on social media is informal and creative, which makes it a hotbed for lexical innovation. Recent work using Twitter data has focused, for example, on the identification of neologisms (Grieve et al., 2018), on their geographical diffusion (Eisenstein et al., 2014), and on trajectories of diffusion (Nini et al., 2017). Empirical investigations on the basis of Reddit data include studies of the linguistic dissemination of neologisms (Stewart and Jacob. 2018) and the role of innovators and adopters (Del Tredici et al., 2018).

The present study is based on Twitter data and goes beyond previous work in its focus on the sociolinguistic dynamics of diffusion, which are at the core of theoretical models of diffusion. Most previous empirical investigations of the spread of new words have been limited to using frequency measures as an indicator of diffusion. While frequency counts have proven useful in previous work, they can only provide limited insight into the sociolinguistic dynamics of diffusion (Stefanowitsch and Flach 2017). In addition to usage frequency, I will therefore use network information to assess the social pathways of diffusion in the present dataset.

## 3 Data

### 3.1 Neologism Sample

The present study is based on a selection of 99 neologisms and investigates their use on Twitter from its launch in 2006 to the end of 2018. The lexemes were selected to cover a broad spectrum of lexical innovation. Previous work by Kerremans (2015, 115–147) has identified four main clusters of neologisms on the conventionalization continuum: ‘non-conventionalization’, ‘topicality or transitional conventionalization’, ‘recurrent semi-conventionalization’ and ‘advanced conventionalization’. The present sample was designed to cover these categories and largely contains neologisms taken from the NeoCrawler (Kerremans et al., 2012), which uses dictionary-matching to retrieve a semi-automatic, bottom-up selection of recent neologisms on the web and on Twitter (Kerremans et al., 2019). I have additionally included several lexemes that were statistically identified to have been increasing in frequency on Twitter in recent years by Grieve et al. (2016). I limit my selection to neologisms whose diffusion started after 2006 to have full coverage of the incipient stages of their spread on Twitter.

### 3.2 Twitter Corpus

Twitter is a popular micro-blogging platform that was started in 2006 and has become one of the most popular social media platforms today. Its broad user base and informal nature allow for a more representative picture of language use than domain-specific studies of, for example, newspaper corpora.^1^ Twitter corpora have been successfully used to identify patterns of sociolinguistic variation in numerous previous studies. A recent study by Grieve et al. (2019), for example, has demonstrated the reliability of large-scale Twitter datasets for studying lexical variation.

Twitter is particularly well-suited for studying lexical innovation due to the scale and types of data it provides, and due to the nature of language use on Twitter. The large size of Twitter’s search index facilitates the quantitative study of neologisms, which requires large-scale datasets due to their inherently low frequency of occurrence. Twitter is widely used to discuss trends in society and technology, which makes it a good environment for studying the emergence of linguistic innovations. The informal and interactional nature of communication on Twitter fosters the rapid adoption of linguistic innovations, and the use of neologisms on social media platforms like Twitter often precedes and drives the diffusion of new words in more formal sources or on the web (Würschinger et al., 2016).

The data for this study were collected using the Python library *twint*, which emulates Twitter’s Advanced Search Function. For each word in the sample, I performed a search query to retrieve all tweets found in Twitter’s search index. Due to the large volume of more frequent lexemes, I limited the sample to contain only candidates for which I could collect all entries found in Twitter’s index. The combined dataset for all 99 lexemes in the sample contains 29,912,050 tweets. The first tweet dates from May 5, 2006 and involves the neologism *tweeter*, the last tweet in the collection is from December 31, 2018, and includes *dotard*.

## 4 Methods

I processed the dataset to remove duplicates, tweets that do not contain tokens of the target neologism in the tweets’ text body. This was mostly relevant in cases where Twitter returned tweets in which the target forms were only part of usernames or URLs.^2^ Hashtag uses were included in the analysis. Retweets were excluded, since the data did not provide reliable information about retweeting activity for the social network analysis. The resulting dataset contains about 30 million tweets, and each tweet contains at least one instance of the 99 neologism under investigation.

To investigate the diffusion of these lexemes in terms of usage frequency, I use time-series of the neologisms’ frequency of occurrence over time. I binned the number of tweets per lexeme in monthly intervals to weaken uninterpretable effects of daily fluctuations in use, and to achieve a reasonable resolution to compare the use of all lexemes, which differ according to their overall lifespan. I visualize the resulting time series as presented in Figure 2.

To capture different degrees of stability vs. volatility in the use of neologisms over time, I calculated the coefficient of variance for all time series. The coefficient of variance $\left(c_{v}\right)$ is a measure of the ratio of the standard deviation to the mean: $c_{v}=\frac{\sigma }{\mu }$ . Higher values indicate higher degrees of variation in the use of a neologism, which is typical of topical use of words such as *burquini*; lower values indicate relatively stable use of words such as *twitterverse*.

To investigate the diffusion across social networks over time, I subset the time series into four time frames of equal size, relative to the total period of diffusion observed for each neologism. I set the starting point of diffusion to the first week in which there were more than two interactions which featured the target lexeme. This threshold was introduced to distinguish early, isolated ad-hoc uses of neologisms by single speakers from the start of accommodation processes during which new words increasingly spread in social networks of users on Twitter. This specific limit was determined and validated empirically by systematically testing different combinations of threshold values for the offset of number of users and interactions among early users. Setting a low minimum level of interactions per week proved to reduce distortions in the size of time windows, and enabled a more robust coverage of the relevant periods of diffusion. For each neologism, I divided the time window from the start of its diffusion to the end of the period covered by the dataset into four equal time slices that are relative to the varying starting points of diffusion for all words in the sample. The starting points of each time frame are marked by dashed vertical lines in the usage frequency plots presented below (Figure 2).

To investigate the social dynamics of diffusion over time, I generated social networks graphs for each of these subsets. Nodes in the network represent speakers who have actively used the term in a tweet and speakers who have been involved in usage events in the form of a reply or a mention in interaction with others. The resulting graphs represent networks of communicative interaction. Communities are formed based on the dynamic communicative behaviour observed, rather than on information about users’ social relations as found in follower–followee networks. This methodology is supported by previous research, which suggests that interactional networks of this kind are better indicators of social structure, since the dynamic communicative behaviour observed is more reliable and socially meaningful than static network information (Goel et al., 2016; Huberman et al., 2008). While users often follow thousands of accounts, their number of interactions with others provides a better picture of their individual social networks, which are much more limited in size (Dunbar 1992).

To construct the networks, I extracted users and interactions from the dataset to build a directed graph.^3^ Nodes in the graph correspond to individual Twitter users, edges represent interactions between users. I captured multiple interactions between speakers by using edge weights, and I accounted for active vs. passive roles in interaction by using directed edges. I assessed the social diffusion of all neologisms quantitatively by generating and comparing several network metrics, and I produced network visualisations for all subsets for more detailed, qualitative analyses.

On the graph level, I rely on the measures of *degree centralization* and *modularity* to quantify the degree of diffusion for each subset. Degree centralization (Freeman 1978) is a graph-level measure for the distribution of node centralities in a graph. Nodes have high centrality scores when they are involved in many interactions in the network and thus play a ‘central’ role in the social graph of users. The degree centrality of a graph indicates the extent of the variation of degree centralities of nodes in the graph. A graph is highly centralized when the connections of nodes in the network are skewed, so that they center around one or few individual nodes. In the context of diffusion, the graph of a neologism tends to have high centralization in early stages when its use is largely confined to one or few centralized clusters of speakers. Diffusion leads to decreasing centralization when use of the term extends to new speakers and communities and the distribution of interactions in the speech community shows greater dispersion.

The normalized degree centralization of a graph is calculated by dividing its centrality score by the maximum theoretical score for a graph with the same number of nodes. This enables the comparison of graphs of different sizes, which is essential for drawing comparisons across lexemes in the present context. The neologisms under investigation differ with regard to their lifespan and usage intensity, resulting in substantial quantitative differences in network size. This needs to be controlled for to allow for an investigation of structural differences of the communities involved in their use.

Modularity (Blondel et al., 2008) is a popular measure for detecting the community structure of graphs. It is commonly used to identify clusters in a network and provides an overall measure for the strength of division of a network into modules. In the social context, this corresponds to the extent to which the social network of a community is fragmented into sub-communities. Networks with high modularity are characterized by dense connections within sub-communities, but sparse connections across sub-communities. In the context of the spread of new words on Twitter, diffusion leads from use limited to one or few densely connected communities to use in more and more independent communities. This is reflected by higher degrees of modularity of the full graph representing the speech community as a whole. Modularity complements degree centralization since it provides additional information about the number and size of sub-communities who use the target words. I rely on the modularity algorithm to perform community detection, and I visualize the eight biggest communities in each graph by colour.

Since modularity is sensitive to the number of edges and nodes in a graph and thus cannot provide reliable results for comparing graphs of different size, I use degree centralization to analyse diffusion over time, and to assess differences in degrees of diffusion between lexemes on the macro-level. Its conceptual clarity and reliable normalization allow for more robust comparisons on the macro-level.

For visualizing network graphs, I rely on the Force Atlas 2 algorithm (Jacomy et al., 2014) as implemented in *Gephi* (Bastian et al., 2009). Force Atlas 2 is a force-directed algorithm that attempts to position the graph’s nodes on a two-dimensional space such that edges should be of similar length and there should be as little overlap between edges as possible. In the present social network graphs, the algorithm places nodes (speakers) closer to each other if they have one or more edges connecting them (communicative interactions in the form of replies and mentions). Attempts to evaluate and compare these visualisations with results obtained from different algorithms such as Multi-Dimensional Scaling and Kamada Kawai showed similar results across methods for parts of the dataset, but could not be used for the full dataset due to the computational complexity involved in the generation of large-size graphs of high-frequency neologisms. Force Atlas 2 is particularly well-suited for handling social networks in big data contexts and has been widely applied in network science approaches to Twitter data (Bruns 2012; Bliss et al., 2012; Gerlitz and Rieder 2013).

To assess and visualize the influence of individual users in the social network, I use the PageRank algorithm (Brin and Page 1998). PageRank assesses the importance of nodes in a network based on how many incoming connections they have. It was initially used to analyse the importance of websites on the World Wide Web, but it is also frequently applied to determine the influence of agents in social networks (e.g., Halu et al., 2013; Pedroche et al., 2013; Wang et al., 2013). In the present context, PageRank assigns higher scores to speakers who receive more incoming replies and mentions, which I visualise by bigger node sizes in the network graphs. To account for varying degrees of strength in the connection between users, I use edge weights for repeated interactions, visualised by the edges’ width in the graphs.

## 5 Results

### 5.1 Frequency-Based Measures of Diffusion

#### 5.1.1 Overall Usage Frequency

As described in Section 2.1, successful diffusion involves an increase in the number of speakers and communities who know and use a new word. The degree of diffusion of new words is often approximated by usage frequency, i.e., by how many times speakers have used a given word in the corpus. The most fundamental way of using this information is to aggregate usage counts and to rely on the total number of uses observed. The underlying assumption is that neologisms that have been used very frequently in the corpus are likely to be familiar to a large group of speakers who have actively produced the observed uses (‘corpus-as-output’) or have been passively exposed to these neologisms (‘corpus-as-input’) (Stefanowitsch and Flach 2017). Aggregating all instances of usage to total counts is taken to represent the total amount of exposure or active usage, indicating the degree of conventionality in the speech community. In the following, I will use this most basic measure of diffusion as a baseline before I zoom in to get a more differentiated picture of the temporal and social dynamics of diffusion.

The present sample of neologisms covers a broad spectrum of usage frequency. Tables 1–4 presents the candidates under investigation in four groups: six examples around the minimum, around the median, and around the maximum total usage frequency observed in the corpus, as well as six words that will serve as case studies in the following sections. These cases reflect a set of prototypical examples of different pathways of diffusion, and I will use these cases to illustrate more detailed characteristics of diffusion before I present the general patterns found for the full sample of neologisms.

**TABLE 1:** Total usage frequency (FREQ) in the corpus. Most frequent lexemes.

Lexeme	FREQ
tweeter	7,367,174
fleek	3,412,807
bromance	2,662,767
twitterverse	1,486,873
blockchain	1,444,300
smartwatch	1,106,906

**TABLE 2:** Total usage frequency (FREQ) in the corpus. Examples around the median.

Lexeme	FREQ
white fragility	26,688
monthiversary	23,607
helicopter parenting	26,393
deepfake	20,101
newsjacking	20,930
twittosphere	20,035

**TABLE 3:** Total usage frequency (FREQ) in the corpus. Least frequent lexemes.

Lexeme	FREQ
microflat	426
dogfishing	399
begpacker	283
halfalogue	245
rapugee	182
bediquette	164

**TABLE 4:** Total usage frequency (FREQ) in the corpus. Case study selection.

Lexeme	FREQ
alt-right	1,012,150
solopreneur	282,026
hyperlocal	209,937
alt-left	167,124
upskill	57,941
poppygate	3,807

The grouping of neologisms on the basis of their total usage frequency presented in Tables 1–4 largely seems to fit intuitions about diverging degrees of conventionality between the frequency-based groups listed in Tables 1–3. Neologisms such as *blockchain* and *smartwatch*, which are probably familiar to most readers, can be assumed to be more conventional than neologisms from the low end of the frequency continuum such as *dogfishing* (‘using a dog to get a date’) or *begpacker* (‘backpackers funding their holidays by begging’).

However, total frequency counts only provide a limited picture of diffusion since they are insensitive to temporal dynamics of usage. Neglecting temporal information about the lifespan and the period of active use of a new word can distort the quantitative assessment of its degree of conventionality in two directions. Firstly, it carries the danger of overestimating the status of words such as *millennium bug* ^4^, whose total usage frequency largely goes back to a short period of highly intensive usage, after which they fall into disuse, become unfamiliar to following generations of speakers, eventually becoming obsolete. Secondly, total counts can underestimate the conventionality of words such as *coronavirus*, which have already become familiar to the vast majority of speakers, but show comparatively moderate total frequency counts, since they have started to diffuse only fairly recently.

Among the most frequent neologisms presented in Table 1, words such as *twitterverse* and *blockchain*, for example, have similar total frequency counts, but differ significantly with regard to their temporal usage profiles. The neologism *twitterverse* has been in use ever since the start of Twitter, while the diffusion of the much younger *blockchain* only started in 2012. Despite its shorter lifespan, *blockchain* accumulated roughly the same number of uses, but shows significantly higher usage intensity in the more recent past, and can be assumed to be familiar to bigger parts of the speech community.

Similar effects are even more pronounced in the remaining groups of neologisms, since words from the lower ranges of the frequency spectrum are typically affected more strongly by temporal variation in their use. In the following sections, I will include temporal information to get a more fine-grained picture of diffusion.

#### 5.1.2 Cumulative Frequency

**FIGURE 1:**
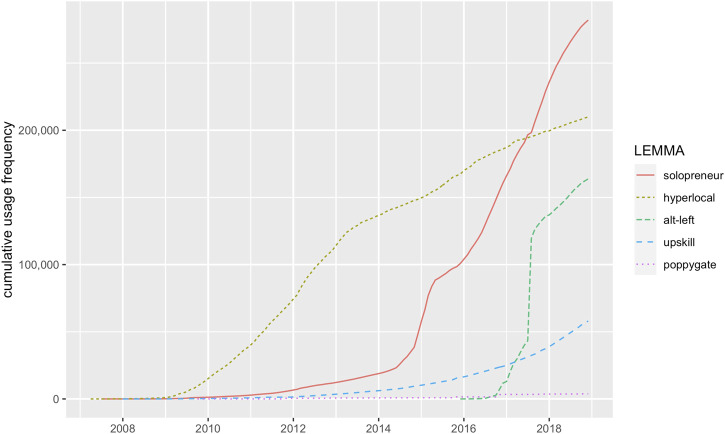
Cumulative increase in usage frequency for the case study lexemes^5^.

Visualising the cumulative increase in usage frequency of new words complements total counts by taking into account the temporal dynamics of their usage intensity over time. Figure 1 presents this information for the case study selection.

While the end points of the trajectories in Figure 1 mark the target words’ total frequency counts as shown in Table 4, the offsets and slopes of the trajectories of usage frequency reveal additional characteristics about differences in their diffusion patterns. The selected neologisms differ regarding their total lifespan observed, which is indicated by diverging starting points of diffusion. The term *hyperlocal*, for example, is the oldest new word among the selected neologisms, and it is commonly used to refer to information that has a strong focus on local facts and events. While it was hardly used in the first years of Twitter, it started to increase in its use in 2009 and was added to the OED’s Third Edition in 2015. Around this time, the neologism *solopreneur* only started to significantly increase in its use. A blend of *solo* and *entrepeneur*, it keeps a low, flat trajectory of sporadic use for about 7 years after its first appearance in the corpus. The first two attestations in the corpus indicate the sense of novelty and scepticism towards the term in its early phases:

1) I’m trying to figure out if I like the term ‘solopreneur’ I just read (July 27, 2007).
2) hmmmmmmm new word added to my vocab = ‘solopreneur’ !! (January 6, 2008).

Most speakers increasingly ‘like the term’ and ‘add them to their vocabulary’ only much later, after 2014, when the phenomenon of individual entrepreneurship attracts increasing conceptual salience in the community, which seems to be both reflected and propagated by the publication of several self-help books for entrepreneurs in this year, which all explicitly use this new term in their titles (e.g., the popular guide *Free Tools for Writers, Bloggers and Solopreneurs* by Banes (2014)). The following short, but intense period of use results in a higher overall number of uses for *solopreneur* as compared with *hyperlocal*, even though the use of the latter term shows a longer lifespan of continual use^5^.

In addition to differences in age, the slopes of the cumulative trajectories in Figure 1 indicate differences regarding the dynamics of diffusion underlying the aggregated total number of uses over time.

Neologisms such as *hyperlocal* and *upskill* (‘to learn new skills’) show a steady, gradual increase in usage frequency over longer periods of time. By contrast, the use of other candidates such as *solopreneur* and *alt-left* is much less stable and less evenly distributed over time.

In the case of *solopreneur*, we observe a big spike in frequency following its increased popularity in the entrepreneurial community in 2014. While it shows the highest total frequency count in Figure 1, the majority of its uses fall into the second part of its observed lifespan.

**FIGURE 2:**
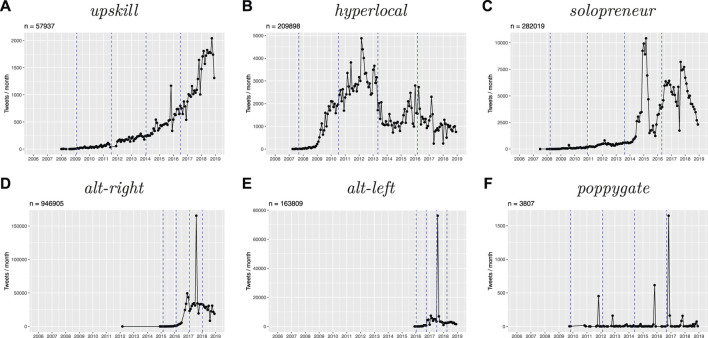
Temporal dynamics in usage frequency for the selected neologisms.

An even shorter and steeper increase can be seen in the use of *alt-left*, which is the youngest neologism to enter the scene at the end of 2015. *alt-left* was coined as a counterpart to the term *alt-right*. The latter neologism is a shortening of *Alternative Right*, introduced by the white-supremacist Richard Spencer in 2010 as a new umbrella term for far-right, white nationalist groups in the United States. Facing substantial criticism for racist attitudes and actions, proponents of this far-right political camp coined and attempted to propagate the derogatory term *alt-left* to disparage political opponents. Despite its late appearance in the corpus, *alt-left* occurs in a total of 163,809 tweets, which places it in the medium range of the sample in terms of total frequency counts. However, its trajectory in Figure 2 shows that the majority of its uses go back to a single period of highly intensive use in the second half of 2017, soon after which it slows down considerably.

The cumulative increase in usage intensity of the selected neologisms illustrates that similar total frequency counts of neologisms can be the product of highly different trajectories of diffusion. These data complement total counts in that they show differences in the total lifespan and in the intensity with which a given neologism was used over time – types of information that are highly relevant for assessing the degree to which they have spread in the speech community.

#### 5.1.3 Usage Intensity

Going beyond cumulative counts, absolute usage frequency counts provide a more fine-grained view of the temporal dynamics of diffusion. Most importantly, analysing usage intensity highlights to what degree new words are being used consistently over time. Figure 2 presents this information for the selected neologisms. In the following section, I will illustrate prototypical differences by referring to the selected cases, before I discuss the results for the full sample^6^.

The absolute frequency plots confirm differences regarding the lifespan and dynamics of usage intensity among the neologisms discussed above. In terms of lifespan, Figure 2 shows that *upskill* and *hyperlocal* are much older than *alt-right* and *alt-left*. The absolute counts also highlight the fact that while there is a low level of use of *solopreneur* since 2007, its main period of diffusion starts much later, in 2014, with a subsequent spike in usage intensity.

#### 5.1.4 Volatility

Besides, the absolute frequency counts over time provide a more detailed picture of the temporal dynamics of use. While the cumulative counts in Figure 1 suggest more gradual trajectories, the plots in Figure 2 indicate that the selected neologisms differ significantly in terms of the volatility with which they are used in the corpus.

The neologism *upskill* shows the smoothest trajectory of diffusion among the candidate neologisms in Figure 2. Aside from two smaller spikes, at the end of 2016 and 2018, it has gradually increased in its use since its first attestation in the corpus at the end of 2007. Neither its frequency counts, nor the corpus data suggest that its spread was triggered or propagated by specific topical events or by the determining influence of individual users or user groups. After a long period of very slow, but consistent increase in frequency, its diffusion has accelerated in recent years. While its future remains uncertain, its previous trajectory resembles most closely the earlier phases of spread as predicted by S-curve models.

While *hyperlocal* also exhibits a marked increase in usage frequency during its earlier stages, its peak in popularity is followed by a decline in use, after which it settles at a relatively stable level of about 1,000 tweets per month. This coincides with the OED’s decision to take up *hyperlocal* in its 2015 edition. Despite fluctuations, *hyperlocal* has been used relatively consistently in the recent past.

The neologism *solopreneur* has been in use since 2007 and shows an overall increase in usage frequency, but its use fluctuates more strongly than that of *hyperlocal*. After its initial peak around 2015, which coincides with the release of several self-help books featuring the term, its frequency plummets, becomes less stable, and shows an overall downward trend.

As was mentioned above, *alt-right* and *alt-left* are closely related. Both terms show high levels of volatility in their usage frequency. The former, older term shows significant diffusion in 2016, particularly in the period leading up to Donald Trump’s election, after which *alt-right* remains in consistent use to a relatively high degree, at about 25,000 tweets per month. Its counterpart, *alt-left*, enters the scene much later, during the infamous Charlottesville Rally in 2017, whose topical effect causes a huge spike in the use of both terms. However, unlike *alt-right*, which reverts to its previous usage intensity, the use of *alt-left* seems to largely disappear from Twitter in the aftermath of the event.

The final example among the selected candidates, *poppygate*, also exhibits high degrees of volatility, and it features the most distinctive pattern of spikes in its usage intensity. Unlike the single topical spike for *alt-right* and *alt-left*, its use follows a recurrent, regular pattern: speakers use it almost exclusively around Remembrance Day, which takes place in November. The term *poppygate* represents a last category of neologisms in the sample, which show strong fluctuations in usage intensity, but for which these patterns follow a regular temporal pattern.

To quantify the degree to which neologisms are used with consistent frequency over time, I calculate and compare the coefficients of variation for each neologism in the sample. This metric captures the overall volatility in usage frequency of words over their lifespan relative to their average frequency of occurrence in the corpus. Tables 5–7 presents the coefficients of variation for the selected neologisms, as well as for the top and bottom six neologisms that show the highest and lowest degrees of variation in the sample.

**TABLE 5:** Coefficients of variation (VAR) for the selected neologisms.

Lexeme	VAR
hyperlocal	0.98
upskill	1.14
solopreneur	1.20
alt-right	1.81
poppygate	4.75
alt-left	5.31

**TABLE 6:** Coefficients of variation (VAR) for the six neologisms with the lowest scores in the sample^6^.

Lexeme	VAR
followership	0.71
lituation	0.72
twitterverse	0.72
detweet	0.74
remoaners	0.76
twittersphere	0.77

**TABLE 7:** Coefficients of variation (VAR) for the six neologisms with the highest scores in the sample.

Lexeme	VAR
upskirting	9.39
youthquake	6.32
alt-left	5.31
birther	5.00
poppygate	4.75
cherpumple	4.69

The results in Tables 5–7 show that the sample covers a broad spectrum of volatility in usage frequency. Among the neologisms that were used the most consistently, i.e., exhibit the lowest degrees of variation, we find words whose frequency-based measures suggested high degrees of conventionality. For example, *twitterverse* is listed among the most frequent neologisms in Table 1 and is also one of the oldest neologisms, with its first attestation in the corpus dating back to December 19, 2006.

By contrast, the group of lexemes that show the highest degree of volatility in usage frequency is comprised of neologisms with lower degrees of conventionality, which are generally less frequent and were coined more recently. Notably, topical spikes play a crucial role in the diffusion processes of all examples in this category: the diffusion of *alt-left* and *birther* ^7^ was promoted by extralinguistic political events, *upskirting* ^8^ and *youthquake* ^9^ were advanced through increased metalinguistic salience after they were added to the OED and awarded Word of the Year 2017 by Oxford University Press. Both *poppygate* and *cherpumple* ^10^ exhibit recurrent topicality, and are typically only used in the contexts of their seasonal relevance in autumn and winter.

The selected neologisms cover the spectrum of volatility in usage frequency found in the full sample of neologisms, and the coefficients of variation represent quantitative measures which reflect the differences in volatility between the selected neologisms visualised in Figure 2 and discussed above. The frequency-based analysis of the three neologisms discussed above demonstrates that usage frequency counts, particularly when combined with an analysis of their underlying temporal dynamics, can help to approximate the spread and success of neologisms to a certain degree. However, the results also point to substantial limitations of frequency-based approaches to studying diffusion.

The present data demonstrate considerable variation in the degrees of diffusion of neologisms with similar frequency of occurrence in the corpus. Total frequency counts alone would predict high degrees of diffusion for neologisms such as *alt-left*, for example. However, its usage history reveals that its use largely goes back to a short period of high usage intensity linked to a specific topical event. The term’s background suggests that it might not have spread far beyond one particular community of speakers. Such potential distortions of frequency-based measures could partly be resolved by in-depth analyses of temporal usage profiles combined with insights from corpus data and extralinguistic events. However, these in-depth analyses of diffusion are not possible through a systematic frequency-based analysis alone, and they cannot be extended to the large-scale study of larger samples of neologisms. Hence it remains unknown to what degree frequency-based metrics adequately capture social pathways of diffusion. In the following section, I will complement the frequency-based approach by social network analyses to get a more differentiated view of the sociolinguistic aspects of diffusion.

### 5.2 Social Networks of Diffusion

As described in Section 4, the social network analysis is based on the interactions between all speakers who have used the neologisms in the sample. Speakers are represented as nodes in the network graph, and interactions between users in the form of replies or mentions are represented as edges. The network structure of the resulting graphs allows analysing the degree to which the target neologisms have diffused in these networks. To monitor diffusion over time, I split the observed lifespan of each neologism into four equally-sized time slices. These time windows are marked by dashed vertical lines in Figure 2. I then generated network graphs for each time window for each neologism in the sample to analyse the individual pathways of diffusion over time and to compare degrees of diffusion between all neologisms in the sample.

#### 5.2.1 Degrees of Diffusion

As discussed in Section 4, I mainly rely on degree centralization as a quantitative measure of diffusion. I consider increasing diffusion to be reflected by decreasing degree centralization of the graph, thus lower values of centrality indicate higher degrees of diffusion across social networks.

For example, the social graph users of a new word shows high centralization in early stages when its use is largely confined to one or few centralized clusters of speakers. When increasing diffusion extends the use of the term to new speakers and communities, the distribution of interactions in the speech community shows greater dispersion, which should be reflected by lower centrality scores for the social network of speakers.

Tables 8–10 report the degree centrality scores for the selected neologisms and for six lexemes with the highest and lowest scores in the sample

**TABLE 8:** Degree centrality scores (CENT) for the selected neologisms; the scores are based on the most recent time slice for each neologism in the corpus.

Lexeme	CENT
upskill	0.0021
hyperlocal	0.0085
alt-right	0.0144
alt-left	0.0238
solopreneur	0.0523
poppygate	0.0566

**TABLE 9:** Degree centrality scores (CENT) for the six lexemes with the lowest scores in the sample; the scores are based on the most recent time slice for each neologism in the corpus.

Lexeme	CENT
baecation	0.0005
fleek	0.0009
ghosting	0.0013
man bun	0.0016
big dick energy	0.0018
twittersphere	0.0020

**TABLE 10:** Degree centrality scores (CENT) for the six lexemes with the highest scores in the sample; the scores are based on the most recent time slice for each neologisms in the corpus.

Lexeme	CENT
rapugee	0.2580
levidrome	0.2373
kushnergate	0.2309
dronography	0.1530
dotard	0.0979
ecocide	0.0922

The neologisms with the lowest scores for degree centrality are also among the most frequent lexemes in the sample. Overall, frequency and centrality generally tend to produce similar results when used to assess degrees of diffusion. This shows usage frequency and social diffusion correlate, as one might expect. Notable deviations exist, however, and will be further discussed in Section 5.3.

Correspondingly, the neologisms with the highest centrality scores rank among the least frequent candidates in the sample. Notable trends among lexemes with high centrality scores are that they tend to be more recent (e.g., *dronography* ^11^) and/or to exhibit high degrees of volatility (e.g., *ecocide* ^12^). Moreover, this group includes political terms such as *Kushnergate* ^13^ and *rapugee* which are controversially discussed on the left and right ends of the political spectrum. For example, *rapugee* is a derogatory term which was coined after sexual assaults by refugees during New Year’s Eve 2015/16 in Cologne, Germany. Previous work has shown that this term was consciously coined and propagated by a closely connected community of far-right activists to disparage refugees, and that its use on Twitter and on the Web has remained largely limited to these communities (Würschinger et al., 2016). This low degree of diffusion is reflected by the low centrality score for *rapugee*.

The following sections use network visualisations to provide a detailed, partly qualitative analysis of the diffusion for the selected cases to illustrate the social dynamics captured by the quantitative measure of centralization as an indicator of diffusion. The examples represent prototypical pathways based on centralization scores. The in-depth analysis of the social dynamics at play is guided by the detection of communities using modularity clustering (Section 4). The algorithm identifies the eight largest communities in each graph, visualised by colour. Moreover, I rely on the PageRank algorithm (Section 4) to assess the importance of users in the network, visualised by node colour. I use manual inspection of user accounts to validate and further investigate the role of these communities and influential users in the selected diffusion processes.

**FIGURE 3:**
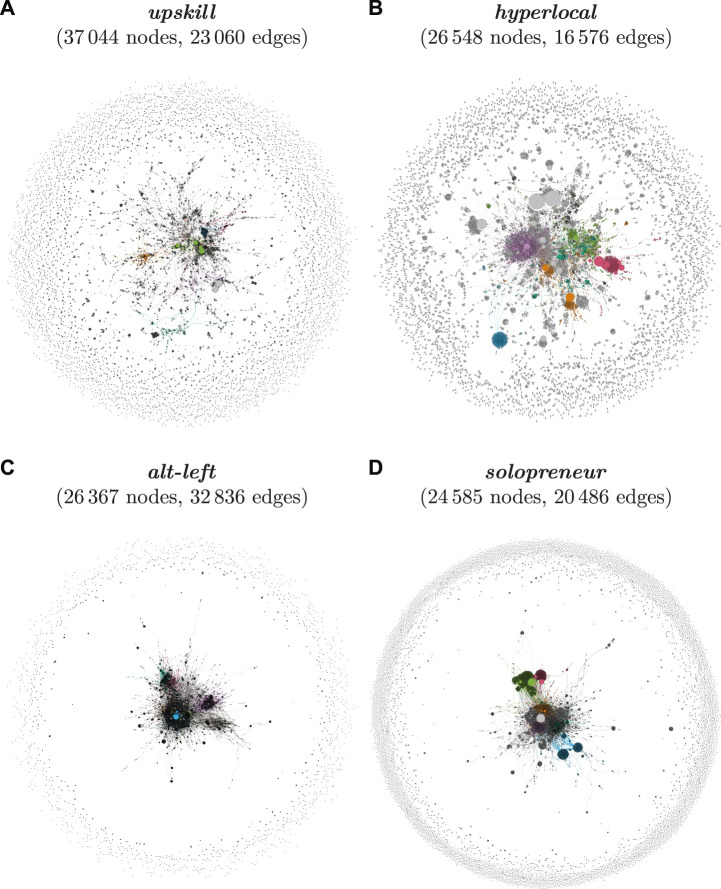
Social network graphs for the last subset of the selected neologisms.

The centrality scores for the selected neologisms cover a broad spectrum of degrees of diffusion, as can be seen in Table 8. Figure 3 presents the full network graphs for four of the selected cases to illustrate differences in the social networks of speakers which are captured by centrality scores.^14^ The network graphs in Figure 3 are sorted according to their degrees of social diffusion–as measured by centrality scores–from (a) to (d). Note that the number of nodes in each graph is very similar, differences between the visualized structure of network graphs are thus due to differences in the underlying social structure of communities rather than a mere function of differences in network size.

The neologism *upskill* exhibits the highest degree of diffusion, which is reflected by the highest degree of dispersion of nodes across the graph in Figure 3A. At the center of the graph, we find a relatively large cluster of speakers who are only loosely connected. Many of these speakers are connected via their affiliations to the world of business, where the term *upskill* is most commonly used. However, on the whole, the use of *upskill* is not limited to a coherent, closely-connected community. The majority of nodes appear towards the fringes and have no connections to the rest of the graph. Speakers use the term independently from each other, without being unified in their motivations to use the term by a common affiliation with a certain community of practice. The social network of *upskill* thus shows an advanced degree of diffusion.

The graph for *hyperlocal* in Figure 3B also shows a high degree of social diffusion, but its use depends more strongly on a central community of users. This core sub-network of speakers forms several smaller clusters which can be linked to certain domains of interest such as journalism, business, and startups, in which the term is most popular. Notably, we observe a stronger role of individual user accounts such as influencers and marketing agencies, which is illustrated by bigger node sizes (representing high PageRank scores). Yet, as in the graph for *upskill*, the majority of occurrences of *hyperlocal* can be traced back to a large number of speakers from a diverse set of sub-communities, which can be interpreted as a sign of advanced diffusion.

The social graph for *alt-left* shows very limited diffusion of the term. Almost all of its use can be traced back to one closely-connected community of users. This core community of users demonstrates typical characteristics of an echo chamber in that it is dense and features strong ties within the community, but has few weak ties connecting it to the rest of the social graph. This observation is in line with the socio-political background of the term, which was coined and propagated by far-right activists in an attempt to unify political efforts (‘*Unite* the Right Rally’) and to distance themselves from and protest against the political left. Inspection of the network reveals that the most influential node in the network is Donald Trump. His use of the term was followed by a sharp increase in usage intensity in the course of the Charlottesville Rally in August 2017. The high degree of social compartmentalization in the use of *alt-left* is also reflected in the ratio between the number of nodes and edges in its graph, which confirms that its community of speakers is much more closely connected than that of the remaining neologisms^15^. Notably, the same applies to the community of *alt-right*, which occupies the opposite pole of the political spectrum. The results for these two terms are in line with previous work reporting effects of political polarization in online social networks for these political communities (Sunstein. 2018). Overall, *alt-left* thus shows a low degree of diffusion. It has received significant popularity in certain parts of the speech community, but its use remains strongly limited to these communities.

Lastly, the social network of speakers using the term *solopreneur* also shows limited diffusion. A significant proportion of its use comes from a diverse set of individual speakers and micro-communities, which are placed at the fringes of the graph. However, similar to the social graph for *alt-left*, a relatively well-connected, large core of speakers is responsible for the majority of its use in the corpus. Moreover, unlike the example of *alt-left*, this central community of users is in turn dominated by the high centrality of a small number of individual accounts. Inspecting the network of users reveals that these ‘influencers’ are all either proud, self-proclaimed solopreneurs, or coaches and agencies that are using the term to promote their services to aspiring entrepreneurs. Overall, *solopreneur* has achieved significant popularity within certain communities, but its use in these communities is unevenly distributed and depends strongly on a small number of individual users. The term does not show signs of advanced diffusion since its use is largely limited to certain individual speakers and communities of practice.

In summary, the social networks of speakers reveal significant differences in the degrees of social diffusion for the neologisms in the present dataset, as observed in the period leading up to the cutoff point at the end of 2018.

While the centrality measures generally concur with the frequency-based analysis of the neologisms discussed in Section 5.1, the network metrics and visualisation add information by providing a more detailed picture of degrees of social diffusion and highlight cases for which the social dynamics of diffusion diverge from what could be observed by relying on usage frequency alone.

#### 5.2.2 Pathways of Diffusion

**FIGURE 4:**
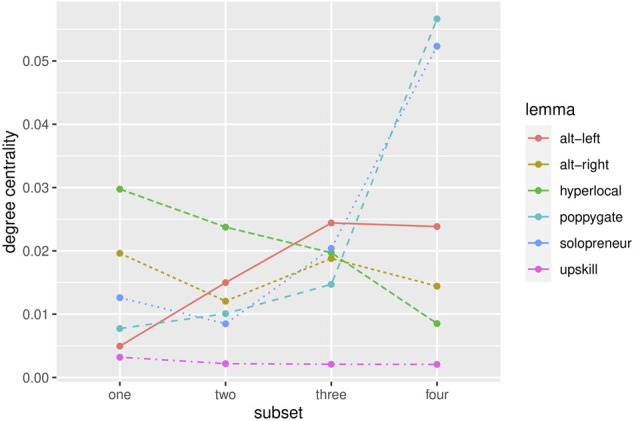
Pathways of diffusion for the selected neologisms. The graph shows DEGREE CENTRALITY scores over time, each SUBSET representing one network graph which was generated for each of the four equally-sized time slices for each neologism in the sample.

To investigate the pathways of social diffusion, Figure 4 presents the degree centrality scores for the selected neologisms over time. The scores for Subset 4 represent the final degrees of diffusion as presented in Table 8. The corresponding network graphs for this stage were presented in Figure 3. The centrality scores for the preceding subsets now add information about the diffusion history of these neologisms. The diverging trajectories of centralization over time indicate significant changes over time as well as differences in the pathways of diffusion between neologisms.

**FIGURE 5:**
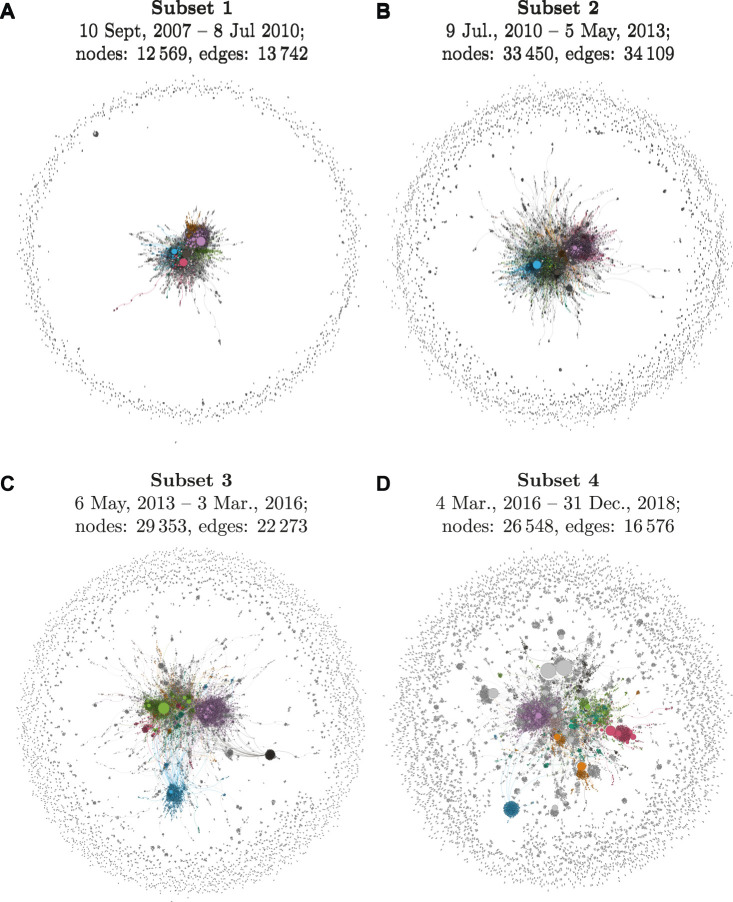
Social network of diffusion for *hyperlocal* over time.

Figure 5 presents the full network graphs for all stages of diffusion for the term *hyperlocal* to illustrate the social dynamics underlying the quantitative measures.

Both the quantitative measure in Figure 4 and the network visualizations in Figure 5 indicate that *hyperlocal* shows increasing, successful diffusion over time. Its use is relatively centralized in its earlier stages, which can be seen from the fact that most speakers who have used the term are closely connected in the social graph in the first quarter of its observed lifespan. Inspecting the most influential speakers and sub-communities in the network (based on PageRank and Modularity scores) reveals that *hyperlocal* is mainly used by a relatively small community of individual journalists in the first subset, who are early adopters in trying to target news to local audiences and use the term very frequently to label this new approach.

In Subset 2, the community of journalists grows and starts to include also bigger news outlets such as *The Guardian*. Additionally, a new community of practice adopts the term: several marketing agencies start promoting their services using the term *hyperlocal*. At this point, the usage intensity of the term peaks, as was demonstrated in Figure 3B. However, the social network data indicate that at this point its use is still mainly the product of high popularity and usage intensity within a small number of dense sub-communities rather than a sign of advanced diffusion across bigger parts of the speech community.

The network graphs show that the social diffusion of *hyperlocal* is only significantly advanced in the last two stages. While we see only few weak ties during the earlier stages of its use, the term now increasingly diffuses beyond its early adopters. Inspecting the network reveals that the use of the term becomes increasingly popular in the world of business and startups as well as the general public on Twitter. The network metrics indicate that individual agents and sub-communities now play a far smaller role in its overall use. While *hyperlocal* shows less usage intensity during these later stages, the network metrics indicate a high degree of diffusion for the second half of its observed lifespan. The timing of its addition to the OED in 2015 supports these observations. The term *hyperlocal* has successfully spread beyond its subcommunities of early adopters, and it seems to be used by a diverse community of speakers from different backgrounds, which renders it a case of advanced diffusion. This process of increasing diffusion for *hyperlocal* is also reflected in its decreasing measures for graph centrality in Figure 4.

The remaining cases in Figure 4 show different pathways of diffusion, both in terms of their overall degree of diffusion and diachronic trajectory. Due to space limitations, I can only provide an overview of their development over time.

Besides *hyperlocal*, the second neologism which exhibits advanced diffusion is *upskill*. In this case, however, we observe little change over time, its degree centrality has been very low since its early attestations in the corpus. This indicates a gradual spread across speakers which is not significantly affected by a small group of influential speakers. The term *upskill* has been used by a wide variety of speakers throughout its observed lifespan and shows the highest degree of diffusion among the selected cases.

By contrast, *solopreneur* and *poppygate* show a negative trend in terms of diffusion. The term *solopreneur* features low degrees of diffusion in its earlier stages, but its use becomes more centralized over time. This is in contrast with its usage intensity over time (Figure 2): while its earlier period of moderate use goes back to a decentralized cluster of users, its increase in usage frequency coincides with a narrowing of its user base. As the network analysis in Figure 3D demonstrates, it becomes increasingly limited to a relatively small community which shares interest in a small professional niche.

The case of *poppygate* exhibits a similar trend towards increasing centralization. Its temporal dynamics show a pattern or recurrent topical usage (Figure 2). The social networks of *poppygate* suggest that while the term was used by a broader audience in its earlier stages, its use in the more recent past goes back to certain communities of speakers for which a specific topical event emerges as a salient occasion to use the term. For example, its most recent spike in usage intensity in November 2016 was caused by a controversy about whether Fifa was right to take disciplinary action against the national teams of England and Scotland after their players wore poppy armbands during a football match between the two nations on 11 November. Protests by the football community caused a spike in usage intensity for *poppygate*, but did not trigger its diffusion beyond this community^17^.

Lastly, *alt-right* and *alt-left* show limited degrees of diffusion over their lifespan. While the centrality of *alt-right* remains fairly stable over time, *alt-left* shows increasing centralization. Both terms are strongly tied to the political discourse surrounding the Unite the Right Rally in the United States and consequently exhibit a sharp increase in usage intensity in the course of the event in August 2017 (Figure 2). This increase in use is, however, reflected by increased centrality scores for both lexemes in Figure 4. This period of highly intense use is thus characterised by relatively smaller rather than larger degrees of diffusion for both lexemes. While the use of *alt-right* reverts to more decentralized use afterwards, the use of *alt-left* remains at this high level of centrality. This seems to confirm the echo chamber effect for *alt-left* discussed in Section 5.2.1: the term has become conventional and popular among a community of like-minded individuals, but its use remains limited to this community. Given the extreme, far-right attitudes and political orientations prevalent in this group, the majority of Twitter users do not want to be associated with this community of users. Since the term *alt-left* has become highly indexical of support and membership of this political camp, very few speakers are willing to adopt and use the term.

In summary, studying the temporal dynamics of social networks highlights changes in the use of neologisms over time and reveals differenct pathways of diffusion in the sample.

### 5.3 Combining Frequency and Network Information

Having applied the frequency-based and the social network approach to assess the diffusion of the present sample of neologism, this section will combine the results obtained from both approaches and show how they complement each other^16^.

#### 5.3.1 Correlations

A first evaluation of the social network approach to diffusion relies on the correlations of degree centrality with the total usage frequency of neologisms, with their volatility, and with their age as observed in the corpus. Table 11 reports the correlation coefficients for these variables.

**TABLE 11:** Correlations of ‘degree centralization’ (CENTRALITY) with the variables total usage frequency (FREQUENCY), coefficient of variation (VOLATILITY), and observed lifespan in the corpus (AGE) for the full sample of neologisms (*n* = 99) using Spearman’s correlation coefficient (Spearman 1961)^17^.

	ρ	*p*
Frequency	−0.44	<0.001
Age	−0.29	0.004
Volatility	0.28	<0.001

Firstly, centrality shows a significant negative correlation with FREQUENCY. This confirms earlier observations in Section 5.2 which indicated an inverse trend between total usage frequency and centrality. More frequent neologisms show on average higher degrees of diffusion, i.e. increase in frequency correlates with wider spread across the speech community. The fact these two central measures for diffusion correlate can be seen as a cross-validation of both approaches. While external data sources would be needed for a more rigorous evaluation, this overall convergence in results suggests that both metrics capture important aspects of diffusion.

Secondly, the AGE of neologisms in the sample shows a significant negative correlation with centrality. As expected, the use of more recent neologisms tends to still go back to more centralized communities, while neologisms with a longer history of use tend to show more advanced diffusion. Unlike frequency counts, which are directly influenced by the temporal usage history of neologisms, the centrality measure is blind to this information. The fact that these age effects are captured by degree centrality supports the usefulness of the social network approach.

Lastly, VOLATILITY shows a significant positive correlation with centrality. Again, this result is in line with expectations. Neologisms such as *poppygate*, whose use exhibits substantial temporal variation tend to show lower degrees of diffusion than neologisms such as *hyperlocal*, whose use is more consistent and less dependent on the topical salience of extralinguistic events.

#### 5.3.2 Deviations Between Centrality and Frequency

**FIGURE 6:**
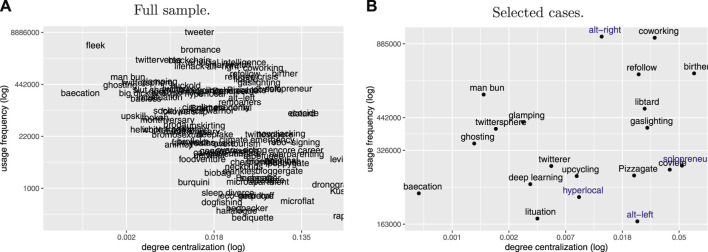
Relationship between total USAGE FREQUENCY and degree centrality (CENTRALIZATION) for the full sample of neologisms (*n* = 99) and the selected cases.

For a closer analysis of the interactions between these variables beyond correlation coefficients, Figure 6 presents all neologisms according to their usage frequency and centrality scores. While Figure 6A covers the full sample, Figure 6B is based on the same data, but zooms in on the frequency range which covers four of the selected cases to provide a clearer view of this section of the sample.

The general trend in the plot confirms the inverse relation captured by the negative correlation coefficient between centrality and frequency. Neologisms with high frequency such as *fleek* have low centrality scores and would thus be assigned a high degree of diffusion by both approaches. The inverse applies to candidates from the lower end of the frequency spectrum such as *microflat*.

However, Figure 6A also shows substantial variation between frequency and centrality scores. Notably, the observed deviations are almost exclusively found towards the right of the diagonal trend, i.e., for cases where centrality assumes lower degrees of diffusion than frequency. For example, while *fleek* and *bromance* are assigned similar scores in terms of their usage frequency, their centrality scores suggest a much lower degree of diffusion for the latter neologism. Similar to cases like *solopreneur* and *alt-left*, which were discussed in detail in Section 5.2.1, centrality thus provides additional information for cases in which the social network structure indicates that the observed usage intensity overestimates the degree of diffusion of a target neologism. This can arise if its observed uses go back to a disproportionately smaller number of speakers and subcommunities.

Analysing these deviations highlights two main groups among the selected neologisms, for which total usage frequency and social network structure seem to diverge in systematic ways^18^. A first group contains neologisms marked by high degrees of volatility in their frequency of use. As shown above, centrality is significantly correlated with volatility. In addition to *poppygate* and *solopreneur*, which were already discussed above, *refollow*, *gaslighting*, *solopreneur*, and *coworking* also show little consistency in their usage. For all of these terms, social diffusion is out of sync with the increase in usage intensity in Figure 6A. It thus seems that the social network approach adds an extra layer of information which comes to the fore especially where frequency-based measures overestimate degrees of diffusion due to the strong impact of short periods of highly intensive use of neologisms in certain parts of the speech community.

A second, converse group with diverging scores contains neologisms whose use is tied to political communities. The neologisms *alt-right*, *alt-left*, *birther*, *covfefe*, *Pizzagate*, and *Kushnergate* are politically controversial and differ strongly in popularity between political camps. It should be noted that these terms also exhibit considerable volatility in their use. Figure 6A shows comparatively lower centrality than frequency scores for these lexemes. Similarly to the cases of high volatility, centrality thus suggests that usage frequency overestimates degrees of diffusion for these cases. While neologisms such as *alt-right* show high frequency counts, the social network analysis reveals that these terms have not spread successfully across communities, and that their use remains limited to certain subcommunities.

#### 5.3.3 Predicting the Success of Lexical Innovations

The results from the network approach show that community structure can be used to assess degrees of diffusion. The social structure of communities during the early stages of diffusion is commonly assumed to be an important factor for the successful spread of linguistic innovations. While a detailed analysis is beyond the scope of the present paper, the present approach yields initial results of the predictive power of social network information.

The dataset shows a significant correlation between the network structure in the first period of diffusion and the overall success of neologisms. Correlating CENTRALITY scores for all neologisms in Subset 1 with their total usage FREQUENCY observed across their full observed lifespan in the corpus yields Spearman correlation coefficient of −0.43 (*p* < 0.001). This means that neologisms are overall more likely to spread successfully if their use is not limited to a centralized network of speakers in their early stages. Among the selected cases presented above, *upskill* fits this pattern: it shows a consistent, successful trajectory of diffusion and its use has been the product of a decentralized bunch of users since its early attestations. Of course, the diverging pathways of diffusion for other words such as *hyperlocal* and *solopreneur* presented in Figure 4 represent exceptions to this general trend. While this trend fits theoretical expectations and the empirical observations in the present dataset, these results remain preliminary. Since centrality correlates with frequency scores, future work based on larger samples, external data for evaluation, and more robust statistical tests is needed to test whether the predictive power of social network features can be confirmed.

## 6 Discussion

In this paper, I have studied the spread of neologisms on Twitter to provide a multi-layered picture of the diffusion of lexical innovations in terms of 1) overall usage frequency, 2) changes in usage frequency over time (volatility), and 3) pathways of social diffusion across members and networks in a larger speech community. The process of diffusion entails social processes which lead to the spread of innovations in social networks (Rogers 1962). Theoretical models characterise the spread of linguistic innovations to new speakers and communities as the key feature of the process of diffusion (Weinreich et al., 1968; Schmid 2020). Despite a broad consensus over the fact that diffusion entails spread in networks of speakers, most previous empirical investigations of lexical innovation have not been based on social network information, but have relied on frequency measures as an indicator for the diffusion of neologisms (Stefanowitsch and Flach 2017). The present study used a large Twitter dataset to investigate the sociolinguistic dynamics of diffusion of neologisms in online social networks. Aside from an in-depth analysis of the spread of neologisms in the present sample, the aim of this paper was to assess the usefulness of using usage frequency and social network data as indicators of diffusion.

### 6.1 Temporal Dynamics of Diffusion

The frequency-based approach revealed that frequency measures can be used to assess degrees of diffusion of lexical innovations with varying success. Total frequency counts (Tables 1–4) proved successful for a coarse-grained distinction between cases of high (e.g., *tweeter*, *smartwatch*), medium (e.g., *monthiversary*, *helicopter parenting*), and low degrees of diffusion (e.g., *begpacker*, *bediquette*). However, differences in the temporal dynamics of use have proved to be necessary for a more accurate assessment of the degrees and pathways of diffusion of neologisms.

Considering the nature of the process and products of *lexical* innovation, this temporal sensitivity is not surprising. Models of linguistic diffusion such as the S-curve model assume competition processes in which several formal variants compete to become the conventional linguistic means to express a certain meaning/function in the speech community. In cases of grammatical innovation, which is at the core of most models and most previous empirical investigations of diffusion, the communicative need for expressing the target concept/function remains stable over time. While grammatical means are, of course, also subject to language change (e.g., *going to*, *will* future), the salience of the target semasiological space (e.g., ‘expressing future intention’), remains stable over time for all speakers in the speech community. Both the direct competition between linguistic variants and the social and temporal invariance of the conceptual space over time are tacit assumptions of S-curve models of diffusion (Blythe and Croft 2012).

Earlier work by Nini et al. (2017) suggests that the diffusion of lexical innovations also follows S-curve trajectories, and the authors use the term ‘semantic carrying capacity’ to refer to the semantic potential of neologisms during diffusion. It seems plausible that the semantic carrying capacity of new words exhibits significant volatility over time and across communities of speakers. While the present study cannot measure or control for changes in semantic potential over time, it tries to account for the temporal sensitivity of neologisms by going beyond cumulated frequency counts and studying their temporal usage profiles.

The present study focused on three main aspects of the temporal dynamics of diffusion: trends in usage intensity, age and volatility. Firstly, trends in usage frequency add information about changes in the degrees of diffusion of neologisms over time. Going beyond total frequency counts, visualising the cumulative increases in usage frequency over time in Figure 1 revealed significant differences in the pathways of diffusion of neologisms with similar total frequency counts. The neologism *hyperlocal* showed the most linear trajectory indicating fairly consistent use, the convex curve of *upskill* indicated a positive trend in its use, and the concave trajectories of *solopreneur* and *alt-left* suggested negative trends in the recent past.

Cumulated frequency counts, which are, in their pure form as total counts, agnostic to temporal trends, have successfully been used as an approximation of the ‘potential exposure’ (Stefanowitsch and Flach 2017) of speakers to linguistic constructions in previous usage-based corpus-linguistic studies. The present results emphasize, however, that temporal trends and changes in usage frequency cannot be neglected when assessing the social diffusion of neologisms, since innovation in the lexicon is subject to high degrees of temporal variation. Notably, trends in usage frequency in the present sample can almost always be traced back to changes in the neologisms’ semantic carrying capacity and are not merely the product of onomasiological competition between formal variants^19^. Typical examples of the influence of topical salience on the use of neologisms are re-current topical neologisms like *poppygate* discussed in Section 5.1.1.

Secondly, it was shown that the age of neologisms provides important information about their diffusion processes. Neologisms such as *hyperlocal* and *alt-left*, which are comparable in total use frequency, but differ strongly with regard to their observed lifespan in the corpus, show different pathways and degrees of diffusion. Older neologisms whose use is distributed more evenly across longer periods of consistent usage (*hyperlocal*) typically show higher degrees of social diffusion than younger neologisms whose use almost exclusively goes back to a short period of highly intensive use (*alt-left*). The positive relationship between the age of neologisms and their degrees of diffusion was supported by the significant correlation with centrality in the network analysis. While a longitudinal, predictive approach to the fate of lexical innovations is beyond the scope of the present paper, it seems possible that neologisms follow Lindy’s Law: the longer new words have been in use in the speech community, the less likely they are to become obsolete in the (near) future (Eliazar 2017). The fate of new words ultimately depends on the conceptual salience of the objects and practices they denote, however: whether *smartwatch* and *blockchain* outlive previous neologisms such as *Walkman* and *Discman* ultimately depends on the future success of these products in our society.

Lastly, the results showed that volatility in use is an important factor in the diffusion of neologisms. While some candidates show fairly consistent usage frequency over time (e.g., *hyperlocal*, *upskill*), most exhibit considerable fluctuations. For some words in the sample, recurrent spikes in usage intensity are an inherent part of their usage profile. The neologism *youthquake* is characterised by spikes in usage intensity when relevant to current public affairs, but shows low frequency of use in the intermediate intervals. Due to the nature of this behaviour, this pattern has been termed ‘topical’ by Fischer (1998). Cases such as *poppygate*, for which these topical spikes occur in fairly regular, periodic intervals, have been classified as ‘recurrent semi-conventionalization’ by Kerremans (2015). For both groups of neologisms total frequency counts cannot provide an accurate estimation of degrees of diffusion since they lack information about these patterns of volatility which are central to these cases of lexical innovation. The network approach to diffusion in Section 5.2 revealed a negative correlation between volatility and degrees of diffusion. It seems that neologisms that are used less consistently over time are less likely to reach advanced degrees of diffusion. Moreover, comparing frequency counts and degree centrality indicated that frequency tends to overestimate the degree of diffusion of topical neologisms. This is in accordance with the observation that isolated spikes in usage intensity tend to go back to disproportionally smaller parts of the speech community.

### 6.2 Social Dynamics of Diffusion

To get a more differentiated view of the social dynamics of diffusion, I conducted a social network analysis of the present dataset. Successful diffusion was defined in Section 2 as spread to new speakers and new communities. Unlike measures such as frequency and volatility which are solely based on the occurrence of neologisms in the corpus, the network approach is based on the social structure of the networks of speakers who have used the target neologisms and thus provides a more direct operationalisation of social pathways of diffusion.

The present results show considerable overlap between frequency and network measures of diffusion. Network centrality significantly correlates with usage frequency, and visualising the relationship between both metrics (Figure 6A) confirms this trend. Both metrics assign high scores for diffusion to established neologisms such as *man bun*, and low scores to less established candidates such as *microflat*. Moreover, centrality shows significant correlations with age and volatility, thus confirming the intuition and general finding that higher usage intensity correlates with wider social diffusion.

The more detailed evaluation of both approaches in Section 5.3.2 also revealed that usage frequency is an imperfect predictor of social diffusion. Centrality generally tends to assign lower degrees of diffusion than frequency for some of the cases in the sample. The main groups affected consist of neologisms whose use goes largely back to specific communities of practice (e.g., *solopreneur*), political communities (e.g., *alt-left*), and/or highly volatile neologisms (e.g., *poppygate*). A closer analysis of these cases in Section 5.2 showed that in these cases the observed number of uses of these neologisms stems from a comparatively smaller number of speakers and communities. It thus seems that the social network information contained in the measure of centrality manages to account for cases in which total usage frequency overestimates degrees of diffusion.

These discrepancies in results reflect two perspective on the process diffusion. Successful diffusion of neologisms was defined as spread to new speakers and new communities. Using the frequency of occurrence of a neologism in a corpus to approximate to what degree it is familiar to bigger parts of the speech community thus has to rely on several assumptions which are only accurate to a certain extent.

Firstly, the number of uses observed might diverge from the number of speakers who are familiar with the term. Frequency can overestimate the latter, for example, if the observed use is the product of high usage intensity by a smaller number of speakers (e.g., *solopreneur*) rather than moderate use by a higher number of speakers (e.g., *hyperlocal*).

Secondly, usage frequency only captures active uses of the term and is blind to the number of speakers who are familiar with the term, but have not used it in the corpus. By contrast, social network metrics also include speakers who have only been passively exposed to the term, and thus covers a broader, and arguably more relevant definition of ‘familiarity’. Network metrics are free from the assumption that the observed output of speakers in the corpus is representative of the input to speakers in the speech community (Stefanowitsch and Flach 2017).

Lastly, the number of uses observed might not be indicative of whether a neologism has spread beyond certain sub-communities and has reached a broader spectrum of the speech community. Many of the neologisms for which centrality indicates significantly lower degrees of diffusion than frequency are socio-politically loaded and known to be used by fragmented and polarized communities, mainly from the far-right end of the political spectrum (Sunstein. 2018). Figure 6B features terms such as *alt-right*, *alt-left*, *birther*, *covfefe*, *Pizzagate*, and *Kushnergate*. Among the selected cases, *alt-left* and *hyperlocal* show a similar total number of uses. Moreover, the numbers of users involved in its use in the last temporal subset are almost identical: 26,367 vs. 26,548. Yet, their social network structure in Figure 3 and their centrality scores indicate far lower degrees of diffusion for *alt-left*. While this political term has become popular among a closely connected community of users, its conventionality remains limited to this social niche and does not extend to bigger parts of the speech community. Its isolated use is in accordance with the socio-linguistic background of the term which was consciously coined by far-right activists as a disparaging out-group term in an attempt to ‘Unite the Right’.

The potential distortions that may arise when assessing the degrees of conventionality of linguistic constructions on the basis of usage frequency alone apply in principle to all linguistic domains. However, the underlying assumptions are particularly problematic in the case of lexical innovation.

Firstly, linguistic *innovations* are by definition new and not (yet) conventional among the speech community. It is therefore to be expected that their use is unevenly distributed across communities of speakers. Since frequency counts alone do not provide information about this distribution, sociolinguistic data are needed to assess the degrees of social diffusion of linguistic innovations.

Secondly, unlike linguistic innovations in other domains such as morphology or syntax, *lexical* innovations are often consciously coined and have a very specific communicative function. Their usefulness is closely tied to the conceptual salience of the entity they denote. The semantic carrying capacity of new words is thus much more likely to exhibit social and temporal variation than the functional potential of grammatical constructions. While speakers of English from all walks of life have felt the urge to talk about the future, the urge to talk about the future of ‘blockchain’ has only come up very recently, is (still) limited to specific parts of the speech community, and might not persist in the future. In other words, the use of lexical innovations exhibits greater social and temporal variation than innovations in other linguistic domains. The interpretation of aggregated frequency counts, which suggest a uniform distribution of use across time and across the speech community, is thus particularly problematic for assessing the diffusion of new words.

Moreover, neologisms typically arise in specific communities of practice and often show, at least initially, high degrees of social indexicality with regard to these communities. The present dataset includes several neologisms which are associated with youth language (*fleek*, *lituation*) and political discourse (*birther*, *alt-left*), for example. A term like *alt-left*, which could in principle be used neutrally to designate the political far-left, is highly socially indexical of the far-right community it emerged from. Therefore it is less likely to be used by speakers outside this community, unless they are willing to be associated with this community. Neologisms which are socially indexical are thus more community-specific. Even when speakers outside this community are familiar with these terms, they are less likely to use them. Usage frequency counts miss such effects, since they only capture active uses of neologisms.

## 7 Conclusion

In summary, the present study has shown that frequency and network-based approaches capture different kinds of information about the use and spread of new words. As we have seen, both approaches show considerable overlap in their overall assessment of degrees of diffusion. On the one hand, measures which are based on the occurrence of neologisms in the corpus such as frequency, age, and volatility capture important aspects about the temporal usage profiles of neologisms. On the other hand, social networks provide a more differentiated view of the social dynamics of diffusion. They allow to visualise and quantify different pathways and degrees of diffusion, which enables a more detailed analysis of the spread of new words to new speakers and communities. While the approaches differ in their strengths and weaknesses, combining information from both approaches provides the most complete picture of diffusion, of course. In corpus-linguistic practice, total frequency counts are the most readily available and most widely used measure for the conventionality of linguistic constructions. The present results suggest that the additional consideration of temporal dynamics of use and social network information can contribute substantially towards a more detailed and accurate picture of diffusion.

As I have argued, the use of network information is of particular importance for the study of neologisms, due to the nature of the process of lexical innovation. However, social network analysis also has great potential for sociolinguistic research in other domains. One of its biggest advantages is that it is usage-based and captures the communicative behaviour of speakers in interaction. It thus enables very fine-grained analyses of the sociolinguistic dynamics of communities, which can be visualised and qualitatively inspected on the basis of network graphs. Additionally, network science offers powerful algorithms to quantify and model the social characteristics of communities on a macro level.

The interactional dynamics discovered by network analyses can be a valuable addition to more traditional, static sociolinguistic information such as metadata about groups of speakers. Moreover, network analyses can be used in cases where metadata about speakers are unavailable, as in the present study. Since the importance of online social networks like Twitter and Reddit is only going to grow in the future, both in terms of their role in society and in academic research, network analyses have great potential for future sociolinguistic research.

## Data Availability

The data analyzed in this study is subject to the following licenses/restrictions: Twitter’s Terms of Service. Requests to access these datasets should be directed to QW, q.wuerschinger@lmu.de.

## Footnotes

1 The present dataset was restricted to tweets in the English language. Due to the absence of the required metadata, the data cannot be further restricted to specific geographical regions, and it is not possible to identify native speakers of English.

2 The post-processing and all quantitative analyses were performed in R Core Team (2018), and the source code is available on GitHub: https://github.com/wuqui/sna.

3 I used several *R* packages (R Core Team 2018) from the *tidyverse* library collection (Wickham et al., 2019) for the network pre-processing; *igraph* and *tidygraph* were used for constructing the networks and for calculating network metrics.

4 The neologisms *millennium bug* was used to refer to ancipated technical problems caused by inconsistent formatting of timestamps at the turn of the century.

5
*alt-right* was omitted from this plot because its high usage frequency would have inhibited the interpretability of the other lexemes; its frequency over time is presented in Figure 3D.

6 Neologisms with a lifespan shorter than 1 year and/or less than 2,000 tweets (*n* = 5) were excluded since the coefficient of variation does not provide robust measures for these infrequent, short-lived outliers.

7 Proponent of the ‘birther movement’, a conspiracy theory which claims that President Obama’s birth certificate was forged and that he was not born in the United States.

8 ‘The habit or practice of taking upskirt photographs or videos’ (OED).

9 ‘A significant cultural, political, or social change arising from the actions or influence of young people’ (https://languages.oup.com/word-of-the-year/2017/).

10 Cherpumple is short for cherry, pumpkin and apple pie. The apple pie is baked in spice cake, the pumpkin in yellow and the cherry in white (https://en.wikipedia.org/wiki/Cherpumple); typically consumed during the holiday season in the US.

11 ‘Dronography is the science, art and practice of creating durable images or video by recording light or other electromagnetic radiation by means of a drone flying around or above a certain scene (Urban Dictionary)’.

12 ‘the destruction of large areas of the natural environment as a consequence of human activity (Merriam Webster Online Dictionary)’.

13 Referring to a political scandal involving Trump’s senior adviser Jared Kushner allegedly meeting Russian officials.

14 The network graphs for *alt-right* and *poppygate* were omitted as their difference in network size does not allow for comparative analyses (*alt-right*: 2,74,686 nodes, *poppygate*: 2473 nodes).

15 The numbers of edges per node for all selected cases in descending order: *alt-right*: 1.49, *alt-left*: 1.24, *solopreneur*: 0.83, *hyperlocal*: 0.62, *upskill*: 0.62, *poppygate*: 0.53.

16 It should be noted that a strict evaluation of both approaches is in principle impossible without external data about the degrees of diffusion for the neologisms under investigation. While such a gold standard for evaluation is inconceivable in the present context, it would be desirable to use additional data sources such as questionnaires, dictionaries or web corpus data for a more rigorous validation of the present approach. This will have to be left for future work.

17 All variables entering the correlation analysis were log-transformed and centred. I report Spearman’s correlation coefficients to avoid assumptions about the linearity of the variables involved. I additionally calculated Pearson’s correlation coefficients, for which the correlation coefficients are slightly higher: FREQUENCY: *ρ* = −0.45, *p* < 0.001; AGE: *ρ* = −0.38, *p* < 0.001; VOLATILITY: *ρ* = 0.23, *p* < 0.001.

18 The present dataset does not allow to assess whether the deviations of the two groups that emerge in this analysis are generalisable.

19 As an exception, the sample contains two sets of formal variants: *monthversary* & *monthiversary* and *rapefugee*, *rapeugee* & *rapugee*.
